# Phenotypic Characterization of Tiger Nuts (*Cyperus esculentus* L.) from Major Growing Areas in Ghana

**DOI:** 10.1155/2020/7232591

**Published:** 2020-08-07

**Authors:** Paul Agu Asare, Roger Kpankpari, Michael Osei Adu, Emmanuel Afutu, Adeyinka Saburi Adewumi

**Affiliations:** Department of Crop Science, School of Agriculture, College of Agriculture and Natural Sciences, University of Cape Coast, Cape Coast, Ghana

## Abstract

Tigernut (*Cyperus esculentus* Lativum) is an important but understudied and underutilized crop in Ghana. The tubers are highly appreciated for their health benefits and nutritive value. To contribute to the conservation process of tiger nut and identify elite genotypes, this study was conducted to assess phenotypic variability in tiger nut genotypes in Ghana. Sixty-four (64) genotypes were collected from major tiger nut growing areas in Ghana. The genotypes were field-grown and characterized based on phenotypic and yield traits. Similarity coefficient (Bray-Curtis) was between 0.82 and 0.98, indicating low variability in both qualitative and quantitative characters. The cophenetic correlation coefficient was 0.64. The genotypes were mainly brown with only a few black (6) tubers from the central region. Materials collected from parts of the eastern region (Aduamoah) generally recorded the highest tuber weight. Tuber weight depended on plant height and number of tillers. There were high tillering genetic materials among the genotypes. Tubers were categorised as oval (10), ovoid (33), or oblong (15). The genotypes clustered into two groups based on shoot and tuber characteristics, rather than on geographical origin. The low genetic diversity among the genotypes suggested either the possible existence of a network among tiger nut farmers in Ghana in circulating the planting material or some form of seed sorting for uniform and homogenous planting materials has been carried out over the years. Our results point to the imperativeness to expand the genetic base of the tiger nuts to facilitate its improvement in Ghana.

## 1. Introduction

Tiger nut (*Cyperus esculentus* Lativum), also known as yellow nutsedge, chufa, earthnut, rush nut, or edible galingale, is a perennial weed of the sedge family widespread globally, particularly, in the tropical and Mediterranean regions of the world [1, 2]. The crop is inaccurately referred to as a nut, but its storage root is a tuber that is produced and consumed fresh, processed, or as a part of medicinal preparations [3]. Tiger nut contains, approximately, 26–30% starch and 21–25% fat, providing about 400–450 kcal 100 g^−1^ energy [2]. It also contains 3–8% protein and 8–10% fibre, in addition to having appreciable proportions of vitamins E and C and some minerals, including phosphorus (P), potassium (K), iron (Fe), and calcium (Ca) [2, 4–10].

The almond-like-sweet tubers, which are rich in energy, protein, fatty acid, and vitamins, could moderate the incidence of colon cancer, coronary heart diseases, obesity, diabetes, and gastrointestinal disorders [2, 8, 10, 11]. The crop is also known to have some aphrodisiac, carminative, diuretic, emmenagogue, stimulant, and tonic effects and is said to be useful in the treatment of excessive stomach gassiness, indigestion, diarrhoea, dysentery, and excessive thirst [4]. The crop is used to treat measles and fever or as a dessert for its sweet flavour in some parts of South America [12], as well as a source of food and perfumes in certain places in North Africa [13].

In Africa, major producers include Nigeria, Niger, Mali, Senegal, Ghana, and Togo. The crop is used primarily uncooked as a side dish or as a vegetable or dried as a sweet snack in most of these countries [14]. In Ghana, tiger nuts are called different names by various ethnic groups with different dialects, including “atadwe” by the Akans, “atangme” by the Gas and “nansaxa” by the Dagombas [15, 16]. Despite being a minor crop, tiger nut is increasingly becoming an important crop in Ghana, due to the recognition it receives by many Ghanaians for its nutritional and health benefits [17]. Among the underutilized crops in Ghana, tiger nut could find many useful applications in the food industry as it is now common to find tiger nut flour used as a thickener, for bread and cakes, or prepared into assorted alcoholic and nonalcoholic beverages and iced creams in Ghana. Estimates suggest that 70% of the farmers of tiger nuts are women [18]. The crop is becoming important for many poor households, which depend on it for income generation and subsistence, as producers and/or traders without the need for large capital investments. Tiger nut could be exploited to provide dietary diversification for the alleviation of micronutrient deficiency, particularly, among the poor and children, as well as contributing to agricultural gross domestic product (GDP) through its local and international trade.

Despite these potential benefits of tiger nut, the crop is still underexploited and underutilized in Ghana. The crop does not make most lists of the so-called neglected and underutilized plant species of the world, perhaps because it is considered as a noxious and invasive weed in many places. Ghanaian farmers still grow unimproved and heterogeneous landraces with poor yield and quality traits. Currently, unlike other crops, including cereals and legumes, and other root and tuber crops, there are no known released varieties of tiger nut in Ghana. There has not been a comprehensive germplasm collection and characterization and there is the likelihood that the valuable local cultivars or landraces of tiger nuts may be lost with time [19]. Besides, documented information on tiger nuts in terms of cropping systems, agronomic practices, and production statistics are scanty; all these limit the possibility of improving tiger nut through breeding and agronomic enhancement.

Strategic collection, characterization, and preservation of genetic resources are essential requirements in crop improvement programs, especially with fresh and underutilized crops, such as tiger nut [20]. Characterization provides the basis for all selection as desired traits are identified and fundamental information about genotypes is provided [19]. Information on the extent and nature of genetic diversity within the germplasm is essential for characterizing and determining the breeding potential of genotypes available to farmers. In the absence of DNA markers, the most available tool for genetic diversity studies is morphological or phenotypic descriptors. Phenotypic characterization facilitates the identification of genotypes with desirable traits for use in crop improvement [21]. Although they could be characterized by huge variabilities and could be prone to some level of subjectivity, phenotypic traits have successfully been used in diversity studies to classify plant genotypes and undertake taxonomic studies [22–27]. Phenotypic characterization consists of recording phenotypic traits that are easily observed and expressed and also highly heritable. It also results in recording the number of traits, which help in selecting genotypes with similar and dissimilar traits [19]. Collection of landraces and systematic characterization into homogenous groups will kick start the process of boosting the productivity of tiger nut in Ghana. The objective of this study was, therefore, to phenotypically characterize and evaluate a wide range of germplasm of tiger nut genotypes collected from major tiger nut growing areas in Ghana. To contribute towards strategic and speed breeding of tiger nut, we employed a cost-effective and rapid procedure to generate baseline information on the morphology and diversity of tiger nut genotypes in Ghana.

## 2. Methodology

### 2.1. Collection of Germplasm

Germplasm of tiger nuts was collected from farmers in major tiger nut growing areas across Ghana. Depending on what was available, dry or freshly harvested matured tubers were collected from four regions of Ghana, namely, Central, Eastern, Brong-Ahafo, and Northern. A total of sixty-four (64) genotypes were collected, and for each genotype, 100 tubers were collected. On collection, each genotype was coded with an alpha-numeric name based on the name of the town and the number of genotypes collected from the said town/village (Supplementary Table 1). The tubers were then put in aerated zip-locked polyethene carrier bags, kept in plastic containers under ambient conditions and carted to the laboratories of the A. G. Carson Technology Village at the University of Cape Coast. Fresh tubers were subsequently air-dried, making sure to preserve the viability of the seeds. All dried seeds were stored at 20°C for two weeks before sowing in the field.

### 2.2. Experimental Conditions and Field Establishment

The study was carried out at the Teaching and Research Farm of the School of Agriculture, University of Cape Coast (UCC; 5° 06 N, 1° 15′ W) between November 2018 and March 2019 under rain-fed conditions. There are normally two seasons of rainfall at the study site. The peak rainfall is in May to June and the minor is in October, with dry periods (harmattan) experienced between November and February. Precipitation, average temperature, and humidity recorded at the site were 773.7 mm, 25.8°C, and 86.3%, respectively. Day length and solar radiation at the experimental site is normally between 11.30 and 12.40 h and 3151 kJ cm^−2^ day^−1^ and 3804 kJ cm^−2^ day^−1^, respectively [28]. The soil was a clayey loam haplic acrisol previously described in Adu et al. [29]. Briefly, the pH of the soil was 6.1 and had 2.2% and 0.5% of organic carbon and total nitrogen (N), respectively. Preexperimental soil analyses also showed that there was 26.1 *μ*g phosphorus (P) g^−1^ and cation exchange capacity (CEC) 6.1 centimoles of charge per kg soil (cmolc kg^−1^) exchangeable potassium (K) [29]. Cassava (*Manihot esculenta* Crantz) had been grown on the site two years before the current experiment, but the site had been lying fallow since then.

**Figure 1:**
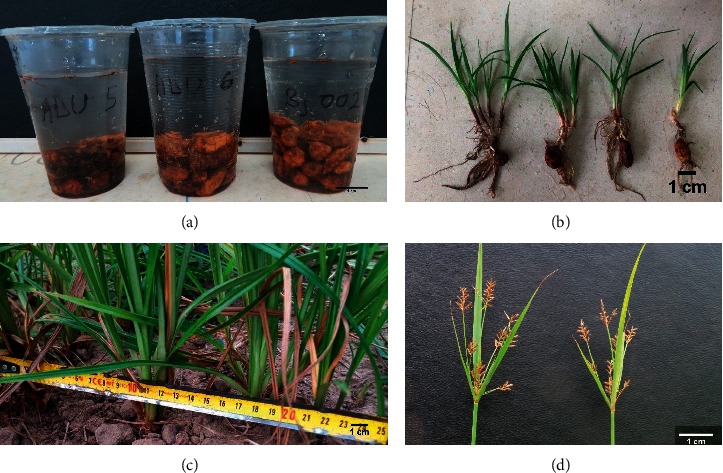
Preparation of tiger nut for planting and some shoot characteristics. (a) Tubers soaked in tap water before sowing; (b) sprout of tubers two days after emergence (DAE); (c) extent of spread of tillers from mother/main plant to the last tiller; (d) flowers of tiger nut.

The land was ploughed and harrowed to a depth of about 30 cm. The experiment was laid out in a completely randomized design with a 0.4 × 4 m plot size for each genotype. To facilitate sprouting, seeds were hydroprimed before sowing and kept for approximately 72 h under ambient temperature (Figure 1). Primed seeds of uniform size of each genotype were manually sown directly into ridged-soil using a wooden seed dibber to a depth of about 5 cm. The tubers were planted on the 28^th^ of November 2018. For each genotype, 20 tubers were planted in single rows of 4 m long. Tubers were excavated at maturity, ranging from 85 to 112 days after planting (DAP). Five plants for each genotype were randomly excavated for data collection purposes, after which the rest were harvested using a hand fork and hoe and the tubers, handpicked from the soil.

### 2.3. Data Collection

The genotypes were characterized based on percentage emergence, number of sprout per tuber at emergence, number of tillers per stand, height at maturity, percentage flowering per genotype, number of tubers per stand, number of rings per tuber, the shape of tubers per genotype, weight of 50 tubers per genotype, tuber colour, and distance from the mother plant to the last tiller. Percentage emergence and number of sprouts at emergence per tuber were assessed soon on the sighting of the first emerged plants, which normally took 5 DAP and was done daily until the 14^th^ DAP when there were no more noticeable sprouts. Percentage emergence was calculated as the quotient of the emerging plants per genotype and the total number of tubers sown and expressed as a percentage. Subsequently, genotypes were categorised as having either low (0–50%), medium (51–79%), or high (80–100%) percentage emergence. The number of sprouts that emerged per tuber of five plants for each genotype was counted to determine the mean sprouting at emergence per genotype (Figure 1(b)). The genotypes were subsequently grouped as having few (1), moderate (2), and profuse (3) sprouts per tuber at emergence. Tillers from five randomly selected plants were assessed weekly from 14 days after sprouting until maturity (where there were no more observed tillers), from each plot, and averaged to compute the number of tillers per plant. The genotypes were then classified as having low (1–9), moderate (10–15) and high (>15) tillers. A measuring steel tape was used to determine the distance from the mother or main stand to the last tiller (Figure 1(c)). The genotypes were grouped as having short (1–5 cm), medium (5–15 cm), long (16–20 cm), and very long (>20 cm) distance between the mother plant and last tiller. At anthesis, the percentage flowering of each genotype was computed. The genotypes were then grouped as no flowers (0%), low (1–15%), medium (16–50%), and high (>50%) flowering genotypes (Figure 1(d)).

**Figure 2:**
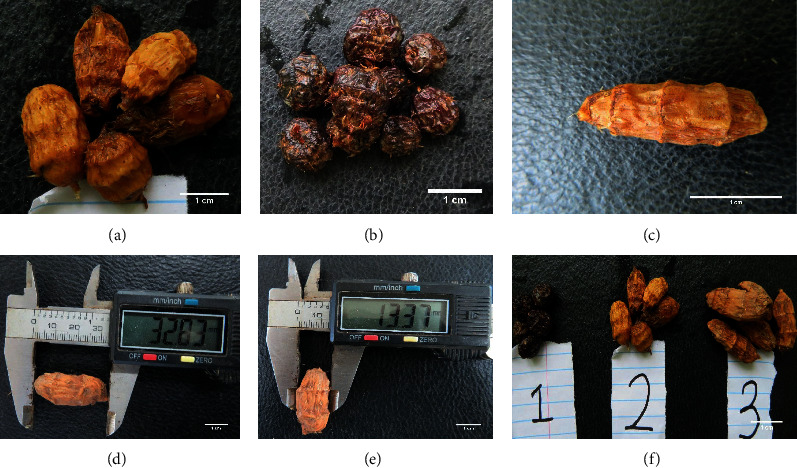
Tuber characteristics after harvest. (a) Brown tuber colour; (b) black tuber colour; (c) tiger nut tuber rings; (d) measurement of the length of tiger nut tuber; (e) measurement of wide of tiger nut tuber; (f) shapes of tubers tiger nut tubers based on length and width ratio (1 (oval), 2 (ovoid), and 3 (oblong)).

At maturity, an average height of five randomly selected plants per genotype was determined, using a measuring steel tape. Plant height was measured from the soil surface to the tip of the longest plumb leaf. The genotypes were grouped as short (1–50 cm), medium (51–75 cm), and tall (>75 cm). Tuber surface colour of the tiger nuts (Figures 2(a) and 2(b)) was determined using a Munsell colour chart. A total of 10 tubers were randomly selected from each genotype and the number of rings counted, and the average was computed. The genotypes were grouped as having low (1–3), moderate (4), or high (>5) number of tuber rings (Figure 2(c)). The shape of the tiger nuts was determined by measuring, with a Venier Callipers to an accuracy of 0.01 mm, the length (L, in mm; Figure 2(d)) and width (W, in mm, considering the section of the tuber with the largest diameter; Figure 2(e)) of ten randomly selected tubers of each genotype. Based on the ratio of L/W, genotypes were considered as oval (<1.3), ovoid (1.3–1.8), or oblong (>1.8) [30] (Figure 2(f)). Tuber number per plant was determined from a sample size of five randomly selected plants for each genotype, and the genotypes were considered as having low, medium, or high tubers if tubers per plant were between 1 and 20, 21–50, or greater than 50 tubers, respectively. Tuber weight was determined by weighing 50 randomly sampled tubers from each genotype using an electronic scale. The genotypes were categorised as having low tuber weight (1–50 g), medium tuber weight (51–100 g), or high tuber weight (>101 g).

### 2.4. Data Analysis

Descriptive and multivariate analyses were employed to analyse the data. Pearson's correlation coefficients for pairs of quantitative traits were calculated at a significance level of 5%. To determine the correlation between the similarity matrix and dendrogram, the cophenetic coefficient was calculated. Classical clustering (paired grouping [UPGMA]) was done using Bray-Curtis similarity efficient to evaluate the similarities or dissimilarities (distance) among the genotypes. The data were processed in Excel and analysed using the GenStat Release 10.3DE, Discovery Edition 4, 2016 (VSN International Limited, Rothamsted Experimental Station, Hemel Hempstead, UK) and paleontological statistics software package for education and data analysis (PAST) [31].

## 3. Results

### 3.1. Qualitative Traits among Tiger Nut Genotypes

While sixty-four genotypes were originally collected (Supplementary Table 1), six genotypes, namely, ASO-008, ASO-019, ADU-009, ADU-025, ADU-028, and ADU-029, did not sprout. Thus, data on fifty-eight genotypes are presented in this paper. Tubers originating from major tiger nut growing areas in Ghana were either oval, ovoid, or oblong (Table 1). The majority (33) of the genotypes had ovoid tubers, followed by oblong (15) and oval (10) genotypes (Figure 2(f)). The majority of the oblong tubers originating from genotypes were collected from Aduamoa in the Eastern Region and were brown (Table 1). The collected tiger nut genotypes were either black or brown with the brown-coloured genotypes (∼90% of total) dominating (Table 1). There were relatively few black genotypes originating from Putobio in the Central region (Table 1).

### 3.2. Variation and Correlation in Quantitative Traits among the Tiger Nut Genotypes

**Figure 3:**
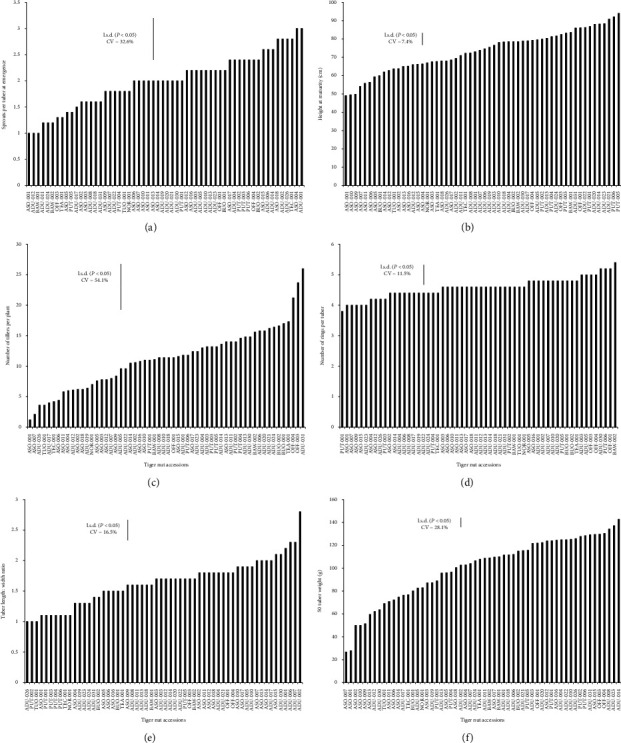
Quantitative traits of tubers of 58 genotypes of field-grown tiger nuts originally collected from major tiger nut growing location in Ghana. (a) Mean number of sprouts at emergence; (b) height at maturity, (c) number of tillers per plant; (d) number of rings per tuber; (e) tuber length and width ratio; (f) 50-tuber weight.

Sprout per plant/tuber ranged from one (ASO-001) to three (ADU-001), with a mean of 3.0 and a CV of 32.6% (Figure 3(a)). Plant heights varied significantly (*p* < 0.05) among the tiger nut genotypes ranging from 42.64 cm (ASO 001) to 92.1 cm (PUT 005) (Figure 3(b)). The number of tillers was significantly (*p* < 0.05) different among the genotypes with a range of 3–26 with a mean of 11 tillers (Figure 3(c)). Similarly, significant differences (*p* < 0.05) were observed in the number of tuber rings among the genotypes evaluated. BAW-002 had the highest mean value of 5.4 while PUT-001 had the lowest (3.8) number of tuber rings (Figure 3(d)). The length/width ratios of tubers (Figure 3(e)) and tuber weight (weight per 50 tubers; Figure 3(f)) varied substantially among the genotypes. Tuber weight ranged from 27.86 9 (A- AFP-001) to 142 g (ADU-0014) (Figure 3(f)).

**Figure 4:**
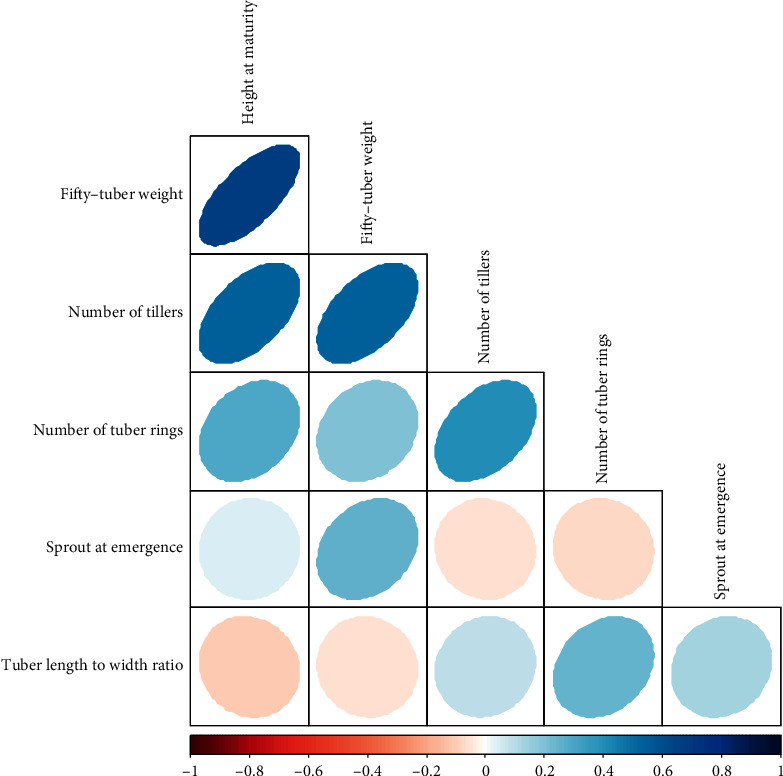
Correlations between quantitative traits observed in field-grown tiger nuts. Eccentricity and colour of circles within the matrix indicate the magnitude of correlation. The scale is indicated in the bar below the matrix greyed-out boxes which indicate nonsignificant relationships (*p* < 0.05).

One-third of all potential correlations were statistically significant (*p* < 0.05) with varying strengths (Figure 4). There were positive and significant correlations between 50-tuber weight and height at maturity (*r* = 0.69); 50-tuber weight and number of tillers (*r* = 0.54); number of tillers and height at maturity (*r* = 0.54). There were also positive and significant correlations between the number of tuber rings and height at maturity (*r* = 0.29) and tuber rings and number of tillers (*r* = 0.41) (Figure 4).

**Figure 5:**
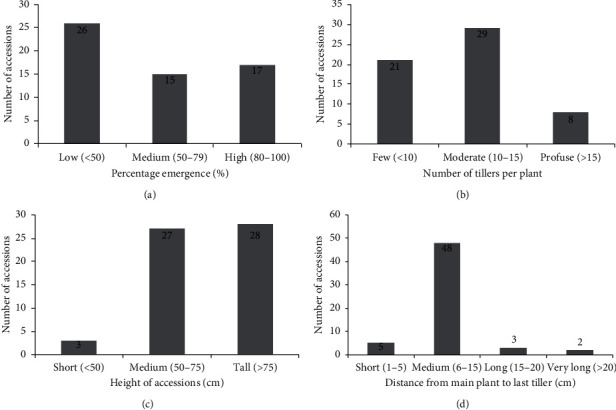
Emergence and shoot characteristics of tiger nut genotypes. (a) Percentage emergence; (b) height of genotype; (c) number of tillers; (d) distance of the last tiller from the main plant to the last tiller.

The percentage emergence for the various genotypes ranged from 0 (ASO-008, ASO-019, ADU 009, ADU-025, ADU-028, and ADU-029) to 100% (ADU-005) with a mean of 47.8% (Figure 5(a)). Emergence was low in half (32) of the genotypes and medium in 24.4% and 26.6% of the genotypes. Few genotypes (∼5%) were classified as short, while there were almost equal proportions of genotypes classified as medium (∼47%) or tall (∼48%) (Figure 5(b)). Out of the 58 genotypes, 50% had 10–15 tillers per plant and 13.8% had greater than 15 tillers per plant, while 36.2% had fewer than 10 tillers per plant (Figure 5(c)). Genotypes that produced brown tubers generally had more tillers than those that produced black tubers. The distance of the last tiller from the main plant varied considerably among the genotypes. The majority (82.8%) of them had the last tillers in the medium distance while 8.6% were at a short distance and 8.6% were at long to a very long distance from the main plant (Figure 5(d)).

**Figure 6:**
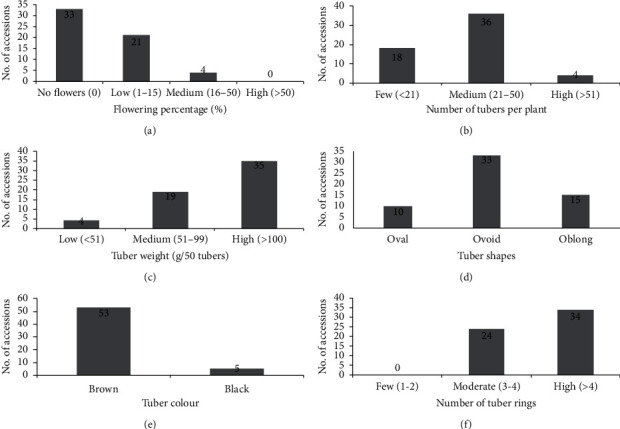
Flowering, number of tubers per plant, 50-tuber weight, tuber shape, tuber colour, and tuber rings of 58 genotypes of tiger nut. (a) Flowering percentage; (b) number of tubers per plant; (c) tuber weight (g/50 tubers); (d) tuber shape; (e) tuber colour; (f) number of tuber rings.

Flowering was very poor among the genotypes collected. More than half (33) of the genotypes did not produce flowers. Twenty-one genotypes (36.2%) produced a few (low) flowers and 6.8% produced a medium number of flowers, whereas none was classified as high-flower producers (Figure 6(a)). The majority of the plants (62.06%) produced 21–50 tubers per plant, while 6.9% (ASO-003, ADU-031 and ADU-010) produced greater than 51 tubers per plant. The rest produced less than 20 or fewer tubers (Figure 6(b)). The majority (60.3%) of the genotypes were categorised as high-tuber-weight producers, with a 50-tuber weight greater than 100g (Figure 6(c)). These genotypes originated mainly from Aduamoa in the Eastern Region. Nineteen genotypes (32.8%) were classified as medium tuber-weight producers, having produced tubers weighing in the range of 51–99 g per 50 tubers. The rest (6.9%) of the genotypes were classified as low tuber-weight producers (Figure 6(c)). Three main tuber shapes (Figure 6(d)), two tuber colours (Figure 6(e)), and two tuber ring categories (Figure 6(f)) were observed.

### 3.3. Cluster Analysis

**Figure 7:**
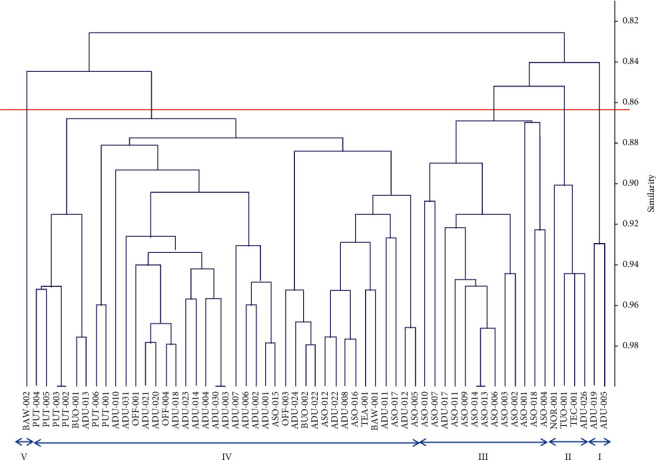
Similarities of tiger nut genotypes based on UPGMA using 11 morphological descriptors and Bray-Curtis similarity coefficients.

The cophenetic coefficient (*r*) value was 0.64. The similarity coefficients for the cluster analysis ranged from 0.82 to 0.98. At 0.83 similarity coefficient, all the genotypes clustered into two main groups and were further divided into five clusters at 0.86 (Figure 7). Cluster I was made up of two genotypes (ADU-005 and ADU-019), which were characterised by medium 50-tuber weight (51–99 g/50 tubers); medium number of tubers per plant (21–50); medium plant height at maturity (50–57 cm); moderate number of tuber rings (3–4) (Figure 7). Four genotypes were grouped within the second cluster. These were brown and oval genotypes which were produced with a low number of tubers and tillers and moderate tuber rings. Cluster III contained 13 genotypes, majority of which were collected from Asokese in Afram Plains and characterized by few numbers of tubers, moderate number of tuber rings, brown tuber colour, and moderate number of sprout at emergence (Figure 7). Cluster IV had the highest number of genotypes (38). In this cluster, both brown and black tubers were found. The black-tuber producers were collected at Putubio in the Central Region. Genotypes in this group produced high tuber weight (>100 g/50 tubers), moderate to high tuber ring numbers (>4 rings), and moderate number (10–15) of tillers (Figure 7). The last cluster was made up of only one genotype (BAW-002), whose tuber was ovoid-shaped and brown-coloured, and it produced a few numbers of tubers with moderate rings and high 50-tuber weight (Figure 7).

## 4. Discussion

There have not been many reports that present the classification of the diversity of tiger nut genotypes or landraces based on phenotypic traits. This study demonstrates that shoots and tubers of tiger nut landraces possess identifiable and distinguishable phenotypic features. The eleven phenotypic traits used in the present study, including plant height, tillers per plant, distance from the mother or main stand to the last tiller, tuber skin colour, number of rings on the tuber, shape of the tuber, and number and weight of tubers per plant, were able to distinguish the 58 tiger nut genotypes into distinct groups. There was variability (high CV) and significant differences (*p* > 0.05) in most of these traits. Observed high variability in percentage emergence among the various genotypes suggests differences in the seed dormancy level of the tubers used for the field establishments. This variability may be controlled by the interaction of genetic diversity and maternal effect, resulting from handling processes before seed collection. Despite this, our results suggest that tiger nut landraces possess distinguishable phenotypic or morphological features that can be exploited through breeding to improve the productivity of the crop.

Most of the genotypes produced a high to a profused number of tillers whilst others produced a low number of tillers, suggesting the presence of high tillering genetic materials among the genotypes. In other grasses, such as rice and wheat, for which the economic yield is from the grains, tillering provides the needed number of stalks for good production and yield and this is controlled by several environmental, genetic factors, and crop management factors [32]. To our knowledge, tillering and its effect on tuber yield in tiger nuts have not fully been exploited, if at all. Perhaps, for root and tuber crops such as tiger nut, the effect of tillering on tuber yield might be explained by the contribution or role of tillers to the photosynthetic capacity and/or radiation-use-efficiency of the plant. Dakogre [19] has noted that higher tillering capacity of tiger nut genotypes suggests that the plants can compensate for any missing stand. What would be interesting, also, would be the relationship between tillering and tuber weight or number in tiger nut. The present results suggest a position correlation between the number of tillers and tuber weight (Figure 4). Moreover, the present results indicate a positive relationship between plant height and quantity of tubers produced, such that genotypes made up of taller plants produced a higher number of tubers than shorter genotypes. This observation corroborates reports that plant height is correlated to biomass production, which makes it a key morphological trait that affects crop yield performance [33]. Most of the genotypes evaluated did not produce flowers because tiger nut is known to be recalcitrant to the production of flowers. It has also been reported that tiger nut flowers rarely appear under field production conditions [13].

Two tuber skin colours, brown and black tubers, were observed in this study. Codina-Torrella et al. [34] previously pointed to the variability in tuber skin colour of tiger nuts and indicated that in some applications, such as the production of some tiger nut drinks and snacks, tuber skins are normally removed before processing. The results here, therefore, confirm those of other authors who have previously reported that tiger nuts consist of two main tuber skin colours [7, 9, 18, 34]. In this study, the majority of the tubers (89.7%) were brown, and, interestingly, all the black tubers were produced by genotypes collected from the Central Region. Many consumers in Ghana who eat, as a snack, the fresh unprocessed tubers, prefer the brown-skinned tubers. It is believed to be more attractive than the black, but the aggregation of the black in a given locality, the Central Region, gives an indication that there is possibly some sort of local preference for the black-skinned tubers in the Central Region, and this is worthy of investigation. Furthermore, there was low variability in the number of rings on the tubers among the genotypes. The number of tuber rings recorded here is consistent with the four to seven rings per tuber reported by Dakogre [19]. Linking the number of rings on the tuber with tuber weight, tubers with more rings were the bigger and heavier in all cases. This positive relationship, if found to be persistent, is suggestive that, in the breeding process, selection for one of the traits will not be detrimental to the other trait and that certain traits could be selected as proxies for others [28].

Tuber weight varied considerably among the genotypes. The present results showed that materials originally collected from Adumoah in the Eastern Region of Ghana produced the highest weight per 50 tubers. Thus, consistent with the results of Codina-Torrella et al. [34], tuber weight varied if tiger nuts were compared according to their origin. Given that the Adumoah tubers were grown in a different agroclimatic region (the Central Region) and yet produced the heaviest tubers, it is plausible that there is a strong genetic component of the variation in tuber weight such that these genotypes have the inherent ability to produce larger tuber size per plant, regardless of the location. Moreover, it is noteworthy that tuber weight correlated with plant height and number of tillers, according to the correlation analysis (Figure 4), suggesting that the increase in plant growth such as plant height and the number of tillers would increase the tuber weight or yield of tiger nuts. Tuber weight did not have a significant relationship with tuber length-width ratio, a tuber shape-related trait (Figure 4), and this was in contrast with the report of Codina-Torrella et al. [34] that showed that elongated tiger nuts were the heaviest. Given that the tuber weight, number of tubers, and length/width ratio are highly heritable [30], these could be exploited in breeding programmes. It could be deduced from the results that consumers prefer brown-coloured tubers to black-coloured ones, which encourages farmers to cultivate more of the brown tuber genotypes compared to black tuber type. Colour will, therefore, be a very important factor to consider in selection.

The majority of the genotypes produced ovoid-shaped tubers indicating that most of the genotypes produce tubers with intermediate tuber length-width ratio. Pascual [30] reported similar findings of three tuber shapes. There was also variability in the number of tubers per plant among the genotypes. The increase in the production of tubers is most likely linked to aboveground vegetative biomass, implying that enhanced aboveground biomass could lead to the production of more tubers [35]. There is also a tendency that tillers may be produced at the expense of tuber formation [18, 19]. The variations in tuber characteristics, including tuber shape, skin colour, weight, and number of tubers per plant, displayed by the genotypes are useful to distinguish between tiger nut germplasms in a programme of genetic improvement even though there was a high similarity among the genotypes studied.

Although the tiger nut genotypes used in the present study were collected from different locations in Ghana, the results suggest that there was low diversity (high Bray-Curtis similarity coefficient of 0.82–0.98) among the genotypes (Figure 6). While tiger nut is a vegetatively propagated material and might be a contributory factor to the observed low diversity, it is also a common practice among subsistence farmers in Ghana to borrow, buy, or exchange seeds or planting materials from each other. Studies from six African countries and covering 40 crops have shown that farmers access over 90% of their seed from informal systems, including own stock, friends, neighbours, and relatives [36]. Dakogre [19] has reported that planting materials of tiger nut are traded by farmers across villages, towns, cities, and borders. The present findings, thus, point to the possible existence of a network among tiger nut farmers in Ghana in circulating the planting material. It is also likely that tiger nut producers in the country have engaged in some level of seed sorting for uniform and homogenous planting materials over the years.

## 5. Conclusions

Based on the phenotypic characteristics evaluated in this study, there is a low diversity of tiger nuts of different geographical origin in Ghana. However, there is the potential to select materials for improved yield. There may be a functional network among tiger nut farmers in Ghana in circulating the planting material, or the planting materials have been homogenised with time through selection by producers. Therefore, tiger nut germplasm in Ghana must be subjected to a long-term prebreeding and genetic enhancement for it to be used in crop improvement programmes. These prebreeding efforts are prerequisites for broadening the genetic base and ultimately exploiting the full potential of tiger nuts in Ghana.

## Figures and Tables

**Table 1:** Shape and colour of tubers of 58 genotypes of tiger nuts grown in the field.

No	Genotype	Tuber shape	Tuber colour
1	ASO-001	Ovoid	Brown
2	ASO-002	Ovoid	Brown
3	ASO-003	Ovoid	Brown
4	ASO-004	Ovoid	Brown
5	ASO-005	Ovoid	Brown
6	ASO-006	Ovoid	Brown
7	ASO-007	Oblong	Brown
8	ASO-009	Ovoid	Brown
9	ASO-010	Oblong	Brown
10	ASO-011	Ovoid	Brown
11	ASO-012	Ovoid	Brown
12	ASO-013	Ovoid	Brown
13	ASO-014	Ovoid	Brown
14	ASO-015	Oblong	Brown
15	ASO-016	Ovoid	Brown
16	ASO-017	Oblong	Brown
17	ASO-018	Ovoid	Brown
18	ADU-001	Oblong	Brown
19	ADU-002	Oblong	Brown
20	ADU-003	Oblong	Brown
21	ADU-004	Oblong	Brown
22	ADU-005	Oblong	Brown
23	ADU-006	Oblong	Brown
24	ADU-007	Oblong	Brown
25	ADU-008	Ovoid	Brown
26	ADU-010	Oblong	Brown
27	ADU-011	Ovoid	Brown
28	ADU-012	Ovoid	Brown
29	ADU-013	Ovoid	Brown
30	ADU-014	Ovoid	Brown
31	ADU-017	Oblong	Brown
32	ADU-018	Ovoid	Brown
33	ADU-019	Ovoid	Brown
34	ADU-020	Ovoid	Brown
35	ADU-021	Ovoid	Brown
36	ADU-022	Ovoid	Brown
37	ADU-023	Ovoid	Brown
38	ADU-024	Ovoid	Brown
39	ADU-026	Oval	Brown
40	ADU-030	Oblong	Brown
41	ADU-031	Ovoid	Brown
42	PUT-001	Oval	Black
43	PUT-002	Oval	Black
44	PUT-003	Oval	Black
45	PUT-004	Oval	Black
46	PUT-005	Oval	Black
47	PUT-006	Oval	Black
48	OFF-001	Oblong	Brown
49	OFF-003	Ovoid	Brown
50	OFF-004	Ovoid	Brown
51	BUO-001	Ovoid	Brown
52	BUO-002	Ovoid	Brown
53	BAW-001	Ovoid	Brown
54	BAW-002	Ovoid	Brown
55	TEC-001	Oval	Brown
56	TEA-001	Ovoid	Brown
57	TUO-001	Oval	Brown
58	NOR-001	Oval	Brown

## Data Availability

The data used to support the findings of this study are available from the corresponding author upon request.

## Supplementary Materials

Supplementary Materials Supplementary Table 1: source and codes of tiger nut accessions collected from major growing areas in Ghana. ER: eastern region; CR: central region; BA: Brong-Ahafo region; NR: northern region.
