# Supplementary material for: Phenotypic Characterization of Tiger Nuts (Cyperus esculentus L.) from Major Growing Areas in Ghana
Source: ScientificWorldJournal. 2020 Aug 7;2020:7232591. doi: 10.1155/2020/7232591 (PMC7428959; doi:10.1155/2020/7232591)
Supplement: Supplementary Materials — Supplementary Table 1: source and codes of tiger nut accessions collected from major growing areas in Ghana. ER: eastern region; CR: central region; BA: Brong-Ahafo region; NR: northern region. [file 7232591.f1.docx]

***Supplementary Table 1:*** *Source and codes of tiger nut accessions collected from major growing areas in Ghana. ER: Eastern region; CR: Central region; BA: Brong-Ahafo region; Northen region.*

| **№** | **Town-Region** | **Accession codes** | **Longitude** | **Latitude** | **№** | **Town-Region** | **Accession codes** | **Longitude** | **Latitude** |
| --- | --- | --- | --- | --- | --- | --- | --- | --- | --- |
| **1** | Aduamoa-ER | ADU-001 | 6°38′9''N | 0°45′8''W | **33** | Asokese-ER | ASO-005 | 6°54′06''N | 0°12′56''W |
| **2** | Aduamoa-ER | ADU-002 | 6°38′29''N | 0°45′24''W | **34** | Asokese-ER | ASO-006 | 6°54′06''N | 0°12′56''W |
| **3** | Aduamoa-ER | ADU-003 | 6°38′28''N | 0°45′26''W | **35** | Asokese-ER | ASO-007 | 6°54′06''N | 0°12′56''W |
| **4** | Aduamoa-ER | ADU-004 | 6°38′20''N | 0°45′4''W | **36** | Asokese-ER | ASO-008 | 6°54′06''N | 0°12′56''W |
| **5** | Aduamoa-ER | ADU-005 | 6°38′34''N | 0°45′30''W | **37** | Asokese-ER | ASO-009 | 6°54′06''N | 0°12′56''W |
| **6** | Aduamoa-ER | ADU-006 | 6°38′9''N | 0°45′8''W | **38** | Asokese-ER | ASO-010 | 6°54′11''N | 0°12′53''W |
| **7** | Aduamoa-ER | ADU-007 | 6°38′10''N | 0°45′9''W | **39** | Asokese-ER | ASO-011 | 6°45′12''N | 0°12′53''W |
| **8** | Aduamoa-ER | ADU-008 | 6°38′31''N | 0°45′29''W | **40** | Asokese-ER | ASO-012 | 6°54′12''N | 0°12′53''W |
| **9** | Aduamoa-ER | ADU-009 | 6°38′13''N | 0°45′12''W | **41** | Asokese-ER | ASO-013 | 6°54′12''N | 0°12′56''W |
| **10** | Aduamoa-ER | ADU-010 | 6°38′21''N | 0°45′7''W | **42** | Asokese-ER | ASO-014 | 6°54′12''N | 0°12′56''W |
| **11** | Aduamoa-ER | ADU-011 | 6°38′21''N | 0°45′7''W | **43** | Asokese-ER | ASO-015 | 6°54′12''N | 0°12′56''W |
| **12** | Aduamoa-ER | ADU-012 | 6°38′9'' N | 0°45′9''W | **44** | Asokese-ER | ASO-016 | 6°54′12''N | 0°12′56''W |
| **13** | Aduamoa-ER | ADU-013 | 6°38′9'' N | 0°45′9''W | **45** | Asokese-ER | ASO-017 | 6°54′17''N | 0°12′53''W |
| **14** | Aduamoa-ER | ADU-014 | 6°38′9'' N | 0°45′28''W | **46** | Asokese-ER | ASO-018 | 6°54′17''N | 0°12′53''W |
| **15** | Aduamoa-ER | ADU-017 | 6°38′19''N | 0°45′23''W | **47** | Asokese-ER | ASO-019 | 6°54′17''N | 0°12′53''W |
| **16** | Aduamoa-ER | ADU-018 | 6°38′19''N | 0°45′23''W | **48** | Tease -ER | TEA-001 | 6°55′51''N | 0°15′20''W |
| **17** | Aduamoa-ER | ADU-019 | 6°38′19''N | 0°45′23''W | **49** | Offinso-ER | OFF-001 | 6°55′20''N | 0°12′40''W |
| **18** | Aduamoa-ER | ADU-020 | 6°38′19''N | 0°45′23''W | **50** | Offinso-ER | OFF-003 | 6°55′18''N | 0°12′37''W |
| **19** | Aduamoa-ER | ADU-021 | 6°38′19''N | 0°45′23''W | **51** | Offinso-ER | OFF-004 | 6°55′18''N | 0°12′40''W |
| **20** | Aduamoa-ER | ADU-022 | 6°38′19''N | 0°45′23''W | **52** | Bawjiase-CR | BAW-001 | 5°40′52''N | 0°33′25''W |
| **21** | Aduamoa-ER | ADU-023 | 6°38′19''N | 0°45′23''W | **53** | Bawjiase-CR | BAW-002 | 5°42′51''N | 0°33′13''W |
| **22** | Aduamoa-ER | ADU-024 | 6°38′19''N | 0°45′23''W | **54** | Putobio-CR | PUT-001 | 5°12′11''N | 1°15′53''W |
| **23** | Aduamoa-ER | ADU-025 | 6°38′19''N | 0°45′23''W | **55** | Putobio-CR | PUT-002 | 5°12′18''N | 1°15′53''W |
| **24** | Aduamoa-ER | ADU-026 | 6°38′19''N | 0°45′23''W | **56** | Putobio-CR | PUT-003 | 5°12′18''N | 1°15′56''W |
| **25** | Aduamoa-ER | ADU-028 | 6°38′19''N | 0°45′23''W | **57** | Putobio-CR | PUT-004 | 5°12′19''N | 1°15′54''W |
| **26** | Aduamoa-ER | ADU-029 | 6°38′19''N | 0°45′23''W | **58** | Putobio-CR | PUT-005 | 5°12′18''N | 1°15′54''W |
| **27** | Aduamoa-ER | ADU-030 | 6°38′19''N | 0°45′23''W | **59** | Putobio-CR | PUT-006 | 5°12′18''N | 1°15′57''W |
| **28** | Aduamoa-ER | ADU-031 | 6°38′19''N | 0°45′23''W | **60** | Tuobodom-BA | TUO-001 | 7°38′34''N | 1°54′39''W |
| **29** | Asokese-ER | ASO-001 | 6°54′16''N | 0°12′54''W | **61** | Techiman-BA | TEC-001 | 7°36′05''N | 1°56′24''W |
| **30** | Asokese-ER | ASO-002 | 6°54′04''N | 0°12′56''W | **62** | Buoyem-BA | BUO-001 | 7°40′07''N | 1°57′50''W |
| **31** | Asokese-ER | ASO-003 | 6°54′06''N | 0°12′56''W | **63** | Buoyem-BA | BUO-002 | 7°40′08''N | 1°57′57''W |
| **32** | Asokese-ER | ASO-004 | 6°54′06''N | 0°12′56''W | **64** | Northern | NOR-001 | 9°35′25’'N | 0°49′06’'W |
